# Gamma Irradiation-Induced Synthesis of Nano Au-PNiPAAm/PVA Bi-Layered Photo-Thermo-Responsive Hydrogel Actuators with a Switchable Bending Motion

**DOI:** 10.3390/polym17131774

**Published:** 2025-06-26

**Authors:** Nikolina Radojković, Jelena Spasojević, Ivana Vukoje, Zorica Kačarević-Popović, Una Stamenović, Vesna Vodnik, Goran Roglić, Aleksandra Radosavljević

**Affiliations:** 1 Vinča Institute of Nuclear Sciences, National Institute of the Republic of Serbia, University of Belgrade, Mike Petrovića Alasa 12-14, Vinča, 11351 Belgrade, Serbia; nnikolina@vin.bg.ac.rs (N.R.); ivanav@vin.bg.ac.rs (I.V.); zokkacpop@gmail.com (Z.K.-P.); una@vin.bg.ac.rs (U.S.); vodves@vin.bg.ac.rs (V.V.); krkljes@vin.bg.ac.rs (A.R.); 2 Faculty of Chemistry, University of Belgrade, Studentski trg 12-16, 11158 Belgrade, Serbia; groglic@chem.bg.ac.rs

**Keywords:** bi-layered hydrogels, gold nanoparticles, PNiPAAm, PVA, photo–thermal energy conversion, soft actuators, gamma irradiation

## Abstract

In this study, we present bi-layered hydrogel systems that incorporate different sizes and shapes of gold nanoparticles (nanospheres and nanorods) for potential use in areas such as photoactuators, soft robotics, artificial muscles, drug delivery and tissue engineering. The synthesized nano Au-PNiPAAm/PVA bi-layered hydrogel nanocomposites provide the unique ability to exhibit controlled motion upon light exposure, indicating that the above systems possess the capability of photo–thermal energy conversion. The chosen synthesis approach is a combination of chemical production of gold nanoparticles (AuNPs) followed by gamma radiation formation of crosslinked polymer networks around them, as the final step, which also allows for sterilization in a single technological step. According to the TEM analysis, the gold nanospheres (AuNSs) with mean diameters of around 17 and 30 nm, as well as nanorods (AuNRs) with an aspect ratio of around 4.5, were synthesized and used as nanofillers in the formation of nanocomposites. Their stability within the polymer matrix was confirmed by UV–Vis spectral studies, by the presence of local surface plasmon resonance (LSPR) bands, typical for nanoparticles of various shapes and sizes. Morphological studies (FE-SEM) of hydrogels revealed the formation of a porous structure with PNiPAAm hydrogel as an active layer and PVA hydrogel as a passive layer, as well as a stable interfacial layer with a thickness of around 80 μm. The synthesized bi-layered photoactuators showed a photo–thermal response upon exposure to irradiation of green lasers and lamps that simulate sunlight, resulting in bending motion. This bending response reveals the huge potential of the obtained materials as soft actuators, which are more flexible than rigid systems, making them effective for specific applications where controlled movement and flexibility are essential.

## 1. Introduction

In the last decade, hydrogel-based actuators, which exhibit controllable shape deformations in response to external stimuli, have been recognized as an innovation in material science with the potential to improve a wide range of applications. The evolution of hydrogel technology has been marked by a shift from conventional rigid actuators to the tremendous flexibility offered by hydrogels [1,2,3,4]. Bi-layered hydrogel actuators, consisting of an active layer and a passive layer, show broad applications in areas such as soft robotics, artificial muscles, drug delivery and tissue engineering due to their inherent flexibility and responses to stimuli. These three-dimensional crosslinked polymer matrices are capable of absorbing and retaining a large amount of surrounding medium, while the introduction of a stimuli-sensitive component as an active layer make them capable of reversible response to external stimuli such as temperature, light, pH, chemicals, etc. [5,6,7,8]. Poly(*N*-isopropylacrylamide) (PNiPAAm), a widely studied thermo-responsive smart polymer, undergoes phase transition around its volume phase transition temperature (VPTT), in the range of 32–35 °C [8,9]. This property allows PNiPAAm hydrogel to serve as an active layer in bi-layered hydrogel nanocomposites, when combined with actuating materials such as metal nanoparticles, carbon nanomaterials, crystal elastomers, etc. [10]. The PVA layer stabilizes the system and improves the hydrogel nanocomposites’ mechanical characteristics, allowing a more effective reversible bending process, and preventing undesirable deformation as well as breaking of the active layer [11]. Noble metals such as silver and gold as nanofillers, among other advanced applications, have been used as photo–thermal energy conversion components due to high photo–thermal conversion efficiency, which is a consequence of the local surface plasmon resonance effect. When the nano-Au/PNiPAAm hydrogel nanocomposite is irradiated with visible light, the photonic energy absorbed by the gold nanoparticles (AuNPs) is rapidly converted into thermal energy, leading to an increase in the temperature of the polymer matrix and its shrinkage. These observations indicate that the investigated systems fulfill the basic requirement (photo–thermal efficiency) for their use as an active layer in photoactuators. This photo–thermal process is an important mechanism for controlled actuation and bending movement. Therefore, AuNPs are an excellent candidate for improving the performance of hydrogel-based actuators [12,13,14]. Gamma irradiation, a technology that has several advantages over traditional approaches, can be employed to synthesize crosslinked polymer structures, without extra chemical crosslinkers, which makes the process cleaner and more effective. In addition, synthesis and sterilization can be completed in a single technical process by employing gamma irradiation as a final production step [8,15,16]. Multifunctional, flexible, wirelessly controlled hydrogel nanocomposite actuators can be used either for soft robotics, bio-applications or both, depending on their properties. This opens up new possibilities for soft wearable applications such as smart surgical devices, intelligent wound management, remote drug delivery systems or other related technologies. The development of soft robotics paves the way for progress, growth opportunities, intelligent design of actively deformable clothing and the creation of soft human–machine interfaces [1,10].

In this study, we investigate the synthesis and characterization of a bi-layer hydrogel nanocomposites consisting of PNiPAAm with AuNPs as the active layer and PVA as the passive layer. The main objective of this work is to investigate the potential of these materials as light-dependent actuators capable of performing a bending motion when exposed to sunlight. The novelty of this research lies in the investigation of the possibility of fine-tuning of photo–thermal response trough the selection of different shapes and concentrations of incorporated gold nanoparticles (AuNPs) within the polymer matrix. This enables a synergistic interplay between the photo–thermal properties of AuNPs and the thermo-responsive behavior of PNiPAAm. Additionally, the use of gamma irradiation as a green and sustainable method for synthesizing the bi-layered hydrogel nanocomposites further highlights the specific advantages of this approach. Potential applications of this technology include soft robots, medical devices and adaptive materials that can respond to environmental stimuli.

## 2. Materials and Methods

### 2.1. Materials

*N*-isopropylacrylamide (NiPAAm), tetrachloroauric (III) acid trihydrate (HAuCl_4_ × 3H_2_O), sodium tetrachloroaurate (III) dihydrate (NaAuCl_4_ × 2H_2_O), sodium citrate (NaC_6_H_7_O_7_), sodium borohydride (NaBH_4_), silver nitrate (AgNO_3_), ascorbic acid (C_6_H_8_O_6_), sulfuric acid (H_2_SO_4_), hydrochloric acid (HCl) and nitric acid (HNO_3_) were supplied by Sigma Aldrich (Hamburg, Germany); cetyltrimethylammonium bromide (CTAB) was purchased from Fluka (Taufkirchen, Germany); and Polyvinylalcohol (PVA, Mw = 72.000 kDa) and 2-propanol (C_3_H_8_O) were obtained from Merck (Rahway, NJ, USA). All substances were commercial products of the highest available purity and were used without further purification. Water from the Millipore Milli-Q system (Millipore Corporation, Cork, Ireland), which corresponds to a purity of four times that of distilled water, was used in all experiments. Prior to the γ-irradiation, the solutions were saturated with high purity (99.5%) argon gas (Messer Tehnogas, Belgrade, Serbia) to remove oxygen. Systems were exposed to gamma irradiation (^60^Co source) in closed cells, under ambient conditions.

### 2.2. Synthesis of Gold Nanospheres and Gold Nanorods

**Figure 1:**
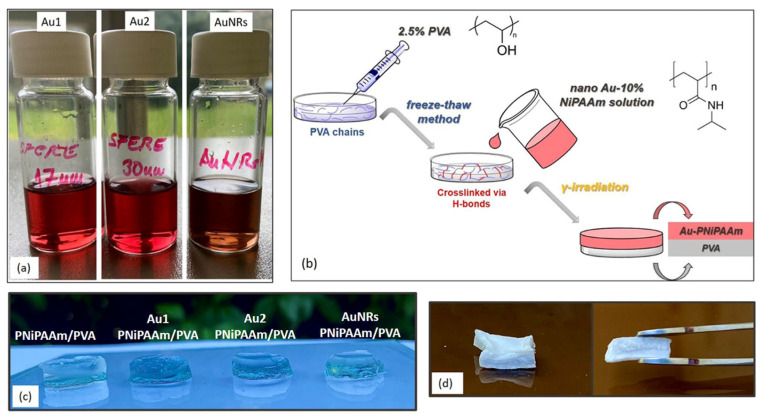
Photograph of colloidal AuNPs (**a**), schematic presentation of the synthesis of bi-layered nano Au-PNiPAAm/PVA hydrogel nanocomposites (**b**), nano Au-PNiPAAm/PVA hydrogel nanocomposites after synthesis (**c**), and lyophilized AuNRs-PNiPAAm/PVA hydrogel nanocomposite (**d**).

Sodium citrate, a well known reducing and stabilizing agent, was used to prepare two colloidal dispersions, named as Au1 and Au2, containing spherical nanoparticles of different sizes [17]. For both colloids, 200 mL of 1 mM Au^3+^ solution was heated and stirred in round-bottomed flasks fitted with reflux condensers. After reaching the boiling point, 20 mL and 10 mL of 38.8 mM sodium citrate were added rapidly for the preparation of Au1 and Au2, respectively. Within a minute, the solutions’ colors changed from yellow to burgundy, indicating the formation of nanospheres (see Figure 1a). The colloidal dispersions were continuously stirred at boiling for an additional 15 min, and then just stirred until cooled to room temperature. The final colloids were centrifuged (10 min, 12,000 rpm), and precipitates were redispersed in 2 mL of water and refrigerated until use.

The two-step procedure was used for the preparation of AuNRs [18]: (1) Au seed formation, and (2) its growth along transversal and longitudinal axes. In the first step, ice-cooled NaBH_4_ aqueous solution (0.01 M, 0.6 mL) was added to a mixture of HAuCl_4_ × 3H_2_O (0.5 M, 5 mL) and CTAB (0.2 M, 5 mL). Yellow-brownish dispersion coloration indicated Au seed formation. For the second step, CTAB (0.2 M, 0.5 mL) was mixed with AgNO_3_ (4 mM, 0.25 mL) and HAuCl_4_ × 3H_2_O (1 mM, 5 mL) solutions. Upon adding ascorbic acid (0.778 mM, 70 mL), when the solution decolorized (from yellow to colorless), 10 mL of Au seed was added. Within 20 min, with gentle stirring at room temperature, the color of the reaction mixture gradually changed from light to dark pink to the final dark violet-blue, indicating AuNRs formation, i.e., seeds’ aging due to their growth along the longitudinal axis (see Figure 1a). In order to remove CTAB excess, to the whole volume of as-prepared AuNRs, 25 mM NaBH_4_ was added and dissolved [18]. After resting for 1 h, the solution was centrifuged (10 min, 12,000 rpm). With supernatant, desorbed CTAB molecules were removed, while precipitate with AuNRs was redispersed in 2 mL of water and refrigerated until further use. The concentrations of Au in the final volumes were determined by inductively coupled plasma–atomic emission spectrometry (ICP-AES), and were found to be 10 mM, 6 mM and 10 mM for Au1, Au2, and AuNRs, respectively.

All prepared nanoparticle samples were labeled as follows: AuNPs refers to gold nanoparticles in general; AuNSs denotes gold nanospheres; Au1 corresponds to nanospheres with a diameter of 17 nm; Au2 refers to nanospheres with a diameter of 30 nm; and AuNRs denotes gold nanorods.

### 2.3. Synthesis of Bi-Layered Nano Au-PNiPAAm/PVA Hydrogel Nanocomposites

The bi-layered systems were prepared by a two-step synthesis method, combining freeze–thaw and radiolytic methods (Figure 1b). In the first step, the freeze–thaw method was used to produce PVA hydrogel as a passive layer (3 repeated cycles of freezing and thawing). An aqueous solution containing 2.5 wt% PVA was poured into a glass mold, frozen at −20 °C for 16 h, and then thawed at 25 °C for 8 h. In the second step, a solution of NiPAAm monomer (10 wt%) was poured over a frozen PVA layer and exposed to gamma irradiation (absorbed dose 25 kGy, dose rate 0.3 kGy/h), under ambient conditions.

To obtain bi-layered nano Au-PNiPAAm/PVA hydrogel nanocomposites (Figure 1c,d), chemically synthesized AuNPs were incorporated into PNiPAAm hydrogel to produce an active layer. The prepared AuNSs (Au1 and Au2) and AuNRs were added to the NiPAAm monomer solutions (10 wt%), poured over the frozen PVA layer and exposed to gamma irradiation to obtain hydrogel nanocomposites. The nano Au-PNiPAAm/PVA hydrogel nanocomposites with two concentrations of both AuNSs and AuNRs were prepared (0.25 mM and 0.50 mM). Moreover, in the case of AuNSs the influence of two different sizes of nanospheres was investigated.

### 2.4. Methods of Characterization

*Gel fraction.* Following the crosslinking process, the hydrogels were sliced and dried at room temperature until they reached a consistent weight. The samples were extracted in distilled water for seven days, with daily water changes to eliminate any unreacted compounds. They were subsequently dried and measured in the same way. Gel content was calculated as:

(1) $$Wg=\frac{m_{ae}}{m_{be}}\cdot 100$$

where *m_ae_* and *m_be_* are the weights of the dry gels after and before extraction, respectively. All measurements were done in triplicate.

*Optical properties*. The absorption spectra of the Au colloidal dispersions and nano Au-(PNiPAAm/PVA) hydrogel nanocomposites were recorded using a Thermo Fisher Scientific Evolution 600 UV–Vis spectrophotometer (Thermo Fisher Scientific, Waltham, MA, USA) in the wavelength range 300–800 nm. The Au colloidal dispersions were recorded in quartz cuvettes (optical path 1 cm), while the nanocomposite samples were recorded in the post-synthesis state by placing the hydrogel nanocomposites (thickness 0.1 mm–0.5 mm) directly in the optical path of the light.

*Field emission scanning electron microscopy (FE-SEM).* The internal morphology of synthesized systems was examined using field emission scanning electron microscopy (FEI Scios2, Dual Beam system, Thermo Fisher Scientific, Waltham, MA, USA). Sample preparation involved initial immersion in distilled water at 25 °C until equilibrium was reached, followed by freezing at −20 °C for a duration of two days. Subsequently, the lyophilization process was carried out using a Martin Christ Freeze-dryer Alpha 1-2 Ldplus (Osterode am Harz, Germany), operating under a vacuum of 0.310 mbar at a temperature of −32 °C for a period of 24 h. Prior to the SEM analysis, the freeze-dried samples were fractured and covered with thin gold layer (around 15 nm) using a LEICA SCD005 nebulizer (Wetzlar, Germany).

*Transmission electron microscopy (TEM).* To determine the size and morphology of the prepared AuNPs, TEM investigation was performed by JEOL JEM-2100 LaB6 instrument operated at 200KV (JEOL company, Peabody, MA, USA). TEM images were acquired with a Gatan Orius CCD camera at 2× binning. A drop of each Au solution was deposited on a carbon-coated copper grid, allowed to dry at room temperature, and then examined under an electron microscope.

*Inductively coupled plasma*–*atomic emission spectrometry (ICP-AES).* The concentration of Au in colloidal dispersion was determined using an ICP-AES Spectroflame 17 at 4 MHz (Spectro-Analytical Instruments, Kleve, Germany). To prepare for ICP analysis, the Au colloidal dispersion samples (0.1 mL of AuNSs and AuNRs) were dissolved individually in 0.9 mL of a concentrated nitric and hydrochloric acid solution.

*Swelling studies.* Swelling of the hydrogel samples was gravimetrically monitored in distilled water, at 25 °C, in proper time intervals. The xerogel discs (diameter ≈ 5 mm, thickness ≈ 1.5 mm) were immersed in water and measured at predetermined time intervals until they attained constant weight. The degree of swelling was calculated according to the following equation:

(2) $$SD=\frac{m_{t}-m_{0}}{m_{0}}$$

where *m_t_* is the weight of swollen hydrogel at predetermined time intervals, and *m*_0_ is the initial weight of the xerogel. Each swelling test was carried out in triplicate, and the results are expressed as mean values.

*Deswelling studies*. In order to perform deswelling kinetics studies, pre-weighted fully swollen hydrogels were immersed in distilled water at 48 °C (above VPTT). The weight of the hydrogels was measured at predetermined intervals during the deswelling process until they approached constants. The water retention (WR) was calculated by the following equation:

(3) $$WR=\frac{m_{t}-m_{col}}{m_{eq}-m_{col}}$$

where *m_t_* weight of deswollen hydrogel at predetermined time intervals, *m_eq_* is the weight of equilibrium swollen hydrogel, and *m_col_* is the weight of the collapsed hydrogel at the end of the deswelling process. All measurements were performed in triplicate.

*Determination of volume phase transition temperature (VPTT).* VPTT values were determined gravimetrically, at temperatures ranging from 12 to 48 °C. After immersion in water at a specific fixed temperature, hydrogels were left to swell to equilibrium and weighed, and the SD_eq_ was calculated at each temperature by using Equation (2). All data were expressed as the average of three independent measurements to ensure reliability.

*X-ray diffraction (XRD).* Gold nanoparticles’ crystalline structure was investigated using a Bruker D8 Advance diffractometer (Bruker, Billerica, MA, USA), with Cu Kα1 radiation at a wavelength of λ = 0.1541 nm. The Cu Kα1 radiation at a specific wavelength provides enhanced sensitivity for crystallographic information, especially for nanoparticles with small unit cells or defined crystal structures. The diffractograms were recorded over the 2*θ* range from 10° to 85°, with an exposure time of 10 s and a 0.05° step increment.

*Fourier Transform Infrared (FTIR) Spectroscopy.* To explore the molecular structure of the hydrogel matrix and its interactions with AuNPs, FTIR analysis was undertaken for both PNiPAAm/PVA hydrogels and the corresponding hydrogel nanocomposites. The analyzed samples were recorded in the xerogel state after being dried to a consistent mass at room temperature. This analysis was performed using a Thermo Electron Corporation Nicolet 380 Spectrophotometer (Thermo Fisher Scientific, Waltham, MA, USA) equipped with the ATR compartment for analysis at room temperature. The spectra were obtained by averaging 64 scans recorded in the range of 4000 to 400 cm^−1^, with a spectral resolution of 4 cm^−1^.

*Mechanical Properties.* The mechanical properties of the PNiPAAm/PVA hydrogels and the corresponding hydrogel nanocomposites were tested using a Universal Testing mechanical analyzer AG-Xplus (Shimadzu, Kyoto, Japan), equipped with a force load cell in the range from 0.01 to 1000 N. Hydrogels were tested in swollen state under ambient conditions. A single compression test was performed on samples with diameter ≈ 10 mm and thickness ≈ 5 mm, with a contact force of 0.02 N and the strain rate of 2 mm/min. At least three samples of each specimen were tested, and the mean values were calculated and presented.

*Photo*–*thermal effect experiments.* The photo–thermal activation of incorporated AuNPs inside the polymer network was carried out using two different irradiation systems. A green light laser pointer with a wavelength of 520 nm and power of 100 mW was used for irradiation of AuNRs-PNiPAAm hydrogel nanocomposite samples, as an active layer. A square-shaped hydrogel nanocomposite with dimensions of 5 mm × 4 mm × 1 mm was irradiated from a distance of 30 cm, at room temperature, to investigate the possibility of the conversion of light to mechanical energy. The second test was done by exposing PNiPAAm/PVA hydrogels and nano Au-PNiPAAm/PVA hydrogel nanocomposites to the lamp irradiation (visible light). In order to demonstrate sunlight-driven actuation, samples in the swollen state were cut into strips with dimensions of 20 mm × 3 mm × 1 mm, and exposed to the radiation effect of an Osram Vitalux lamp, which simulates solar irradiation (300 W, white light: UVB (280–315 nm); radiated power is 3.0 W; UVA (315–400 nm) radiated power is 13.6 W; the rest is visible light and IR). The distance between the light source and the samples was kept constant at 30 cm, and the illumination angle was 90°. To ensure that the mechanical movement was caused by the light activation of AuNPs within the active layer and to prevent the samples from heating by the lamp, a water-filled glass dish with a flat bottom was used as a barrier between the samples and the light source.

## 3. Results and Discussion

### 3.1. Optical Properties of AuNPs and Bi-Layered Nano Au-PNiPAAm/PVA Hydrogel Nanocomposites

**Figure 2:**
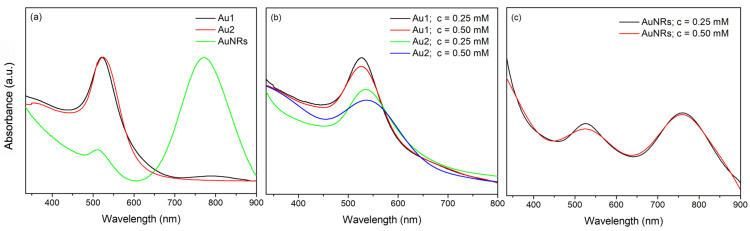
UV–Vis absorption spectra of Au colloidal dispersions (**a**), AuNSs-PNiPAAm/PVA (**b**) and AuNRs-PNiPAAm/PVA (**c**) hydrogel nanocomposites.

In order to test the influence of AuNPs with different sizes and shapes on the features of bi-layered nano Au-PNiPAAm/PVA hydrogel nanocomposites, AuNSs were synthesized by reduction of Au^3+^ ions with sodium citrate as a reducing and stabilizing agent, while the key challenge to form AuNRs was their anisotropic growth, i.e., their preferential growth along longitudinal axis, forming elongated structures. This was accomplished through the addition of CTAB, a surfactant that promotes oriented nanoparticles’ growth [17,18]. The UV–Vis spectra of the thusly prepared AuNPs revealed the characteristic LSPR bands at around 522 nm and 532 nm for nanospheres in Au1 and Au2 colloids, respectively, and two bands at around 510 nm and 770 nm for AuNR solution (Figure 2a). The absorption spectra of the obtained colloids were recorded periodically over six months (not presented here) and indicated that all AuNP solutions remained stable for an extended period.

Bi-layered PNiPAAm/PVA hydrogels were obtained through a two-step procedure. The first step involved crosslinking of PVA by the freeze–thaw technique, in which a crosslinked structure was formed as a result of freezing and subsequent thawing, thus allowing the formation and transition of polymer chains to form a gel [19,20]. After three freeze–thaw cycles, a physically crosslinked PVA hydrogel was successfully formed. It should be noted that, despite the fact that the number of cycles for PVA crosslinking by this technique is typically higher in the literature (ranging from 5 to 8), the number of cycles in this study is lower for two reasons. Firstly, to achieve a stable two-layer system and a strong chemical link between the two layers, the NiPAAm monomer solution must partially penetrate inside the PVA layer after being poured onto it. Secondly, in the next step of the synthesis, the PVA layer is exposed to gamma irradiation, during which it is additionally crosslinked to form a stable passive layer.

On the other hand, to prepare an active layer of the nano Au-PNiPAAm/PVA hydrogel nanocomposites, NiPAAm monomer solutions, containing the appropriate amount of previously prepared solutions of AuNSs (Au1 and Au2) and AuNRs, were poured over the frozen PVA layer and exposed to gamma irradiation to obtain a stable bi-layered structure. The effect of ionizing radiation on aqueous solutions of monomers and polymers leads to polymerization and chain crosslinking through a free radical mechanism, with the degree of crosslinking driven by factors including polymer type, polymer composition, dose rate, total absorbed dose, etc. [8,15]. The UV–Vis absorption spectra of the nano Au-PNiPAAm/PVA hydrogel nanocomposites revealed the characteristic LSPR bands for nanospheres at around 526 nm and 536 nm for Au1 and Au2, respectively (Figure 2b), and two bands at around 525 nm and 760 nm (transverse and longitudinal oscillation, respectively) for AuNRs (Figure 2c).

It is well known that the LSPR of metal nanoparticles represents the collective oscillations of their surface conductive electrons, caused by interaction with electromagnetic radiation. During irradiation, the dislocation of metal electrons in the nanoparticles results in the formation of electric dipoles for small nanospheres or higher order multipole plasmon modes for anisotropic nanoparticles (transverse, longitudinal, tripoles, quadrupoles, pentapoles, etc.), leading to the creation of an additional electric field on the nanoparticle’s surface. The numerous factors, such as the size and shape of the nanoparticles, the aspect ratio in the case of nanorods, dielectric properties of the metal, dielectric permittivity of the environment, inter-particle distance, etc. can influence the LSPR, affecting the movement of conduction electrons within the nanoparticles and thus allowing tuning of the LSPR wavelength region [21,22,23]. Figure 2b clearly illustrates the dependence of the LSPR position on nanoparticle size. A slight red shift of the LSPR from 526 nm to 536 nm with increasing nanosphere size from 17 nm to 30 nm is evident. Furthermore, the plasmon bandwidth (full width at half maximum, FWHM) reflects the nanoparticle size homogeneity, i.e., particle size distribution (PSD). Namely, absorption bands of larger AuNSs have somewhat higher FWHM values, indicating a slightly wider PSD range, which is confirmed by TEM analysis.

However, since the incorporated nanoparticles were exposed to gamma irradiation during the crosslinking process, the potential influence of high-energy ionizing radiation on the properties of the nanoparticles must also be considered. The effects of gamma irradiation on the stability of nanoparticles, including modifications to their size, surface reactivity, thermal stability, and physicochemical characteristics, have been the subject of several studies. For example, previous studies found that nanoparticles withstood both low and high doses of gamma irradiation delivered from a low-energy source (or low dose rate). The National Institutes of Standards and Technologies (NIST) reported that citrate-stabilized AuNPs (as nanoparticle reference materials, RM 8011, 8012, and 8013) did not change their physicochemical properties (including size) after sterilization with 32 kGy γ-irradiation [24,25,26]. The results undoubtedly demonstrate that AuNPs incorporated into the bi-layered nano Au-PNiPAAm/PVA hydrogel nanocomposites maintain their stability with predefined shape and size even after exposure to gamma irradiation.

### 3.2. The Effectiveness of the Chosen Synthesis Methodology

To verify that the chosen combination of freeze–thaw and gamma irradiation synthesis methods was appropriate and effective for polymer crosslinking and formation of a stable bi-layered structure, both the amount of the unreacted residuals after polymer crosslinking and the gel fraction were determined. Following synthesis, the samples were extracted using distilled water for seven days, with the water being changed daily, to determine the quantity of unreacted substances. After the evaporation of the collected water, the mass of unreacted substances was immeasurable, implying that crosslinking was complete. Furthermore, the gel fraction, calculated by Equation (1), was found to be in the range from 97.6% to 98.5% for all synthesized samples. According to the literature, all values above 90% indicate good crosslinking yield [16]. Considering that the produced hydrogels have a bi-layered structure, it is important to note that the layers remained connected even after the samples were immersed in water during the aforementioned experiment.

### 3.3. Morphological Properties of the Nanoparticles and Polymer Networks

**Figure 3:**
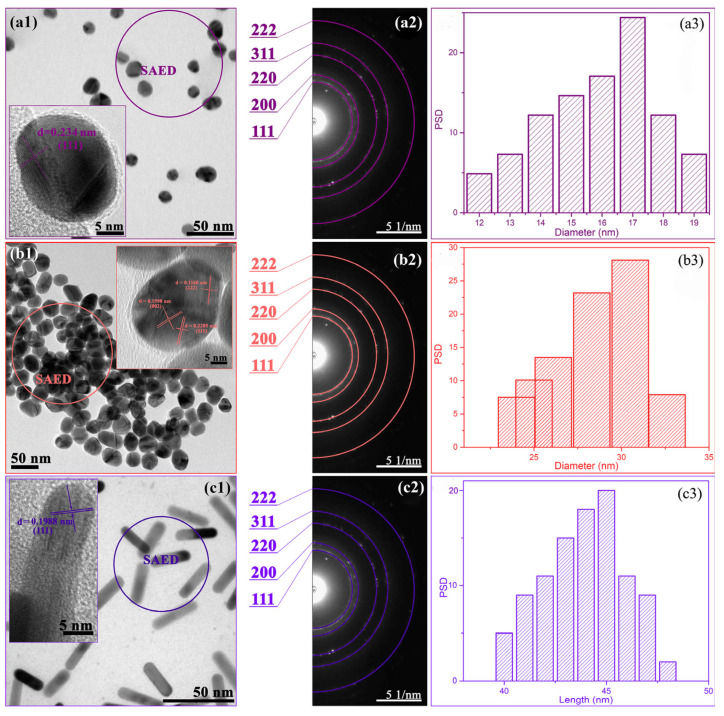
Microscopic analysis of AuNPs: Au1 (**a**), Au2 (**b**) and AuNRs (**c**). TEM micrographs with HRTEM images as insets (**a1**,**b1**,**c1**), corresponding SAED patterns (**a2**,**b2**,**c2**) and particle size distribution histograms (**a3**,**b3**,**c3**).

**Figure 4:**
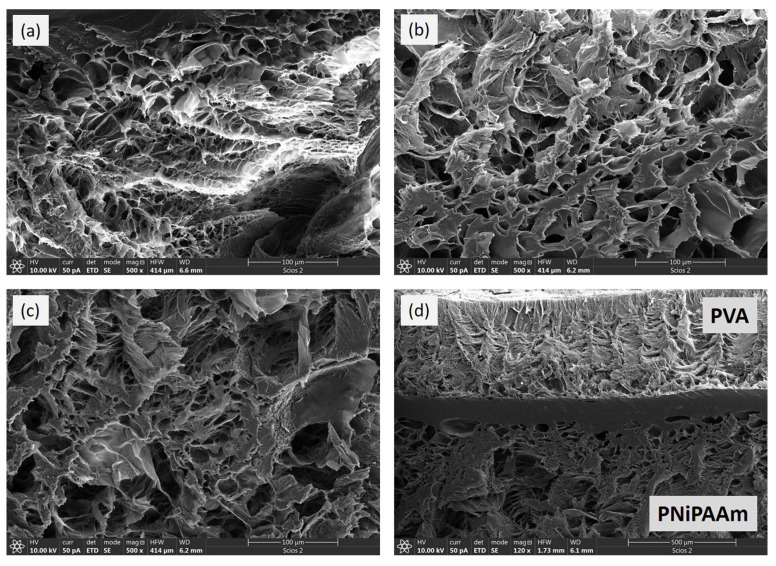
FE-SEM micrographs of PNiPAAm layer (**a**), PVA layer (**b**), AuNRs (0.5 mM)-PNiPAAm layer (**c**) and bi-layered PNiPAAm/PVA hydrogel (**d**).

The structural and morphological properties of the prepared AuNPs, as well as the crosslinked polymer networks, were examined by TEM and FE-SEM analysis, respectively (Figure 3 and Figure 4).

According to the TEM analysis (Figure 3(a1,b1)) and PSD histograms (Figure 3(a3,b3)), dispersion solutions Au1 and Au2 contain spherical nanoparticles with average diameters of around 17.0 ± 2.6 nm and 30.2 ± 2.5 nm, respectively. One may say that citrate ions control particle size, since the different added citrate volumes determined their diameters, i.e., a smaller amount of reducing agent produced larger nanoparticles, allowing them to grow freely to their final size. In addition, HRTEM images of both nanospheres (Au1 and Au2) in Figure 3(a1,b1, insets), revealed crystallographic planes characteristic for face-centered cubic (*fcc*) gold structure [18]. The presence of these planes, and several more, was confirmed in selected area electron diffraction (SAED) patterns (Figure 3(a2,b2)). Similarly, for AuNRs (Figure 3(c1)), the *fcc* gold structure was also recognized (Figure 3(c2)), while their PSD histogram (Figure 3(c3)) indicated the formation of finely dispersed nanorods with approximately 45 nm in length and with an aspect ratio of around 4.5.

FE-SEM cross-sectional analysis of previously lyophilized hydrogel samples was used to evaluate the internal morphology of the investigated systems, and typical micrographs are presented in Figure 4a–d. As expected, both PNiPAAm and PVA layers possess a porous crosslinked structure with similar morphology: micrometer-scale pores with smooth and non-porous interconnected walls [16,23,27,28]. A comparison of PNiPAAm and PVA layer micrographs (Figure 4a and Figure 4b, respectively) reveals that, despite similar morphology, the PVA layer has slightly thicker walls between the pores and a less uniform distribution of pore sizes.

Figure 4c confirms that the presence of AuNPs does not affect the formation of the hydrogel’s crosslinked structure. Due to imaging techniques and equipment limitations, the nanoparticles are not visible in the micrographs [28]. On the other hand, FE-SEM analysis revealed that the two layers formed an interpenetrating network during the synthesis procedure, resulting in an interfacial layer with a thickness of approximately 80 μm. The formation of the interfacial layer results from the permeation of the NiPAAm solution into the PVA network just prior to the second synthesis step, which involves gamma irradiation-induced crosslinking. In addition, the two layers remain connected and intact even after seven days of standing in water during the extraction procedures, but also during the investigation of the photo–thermal effect and the bending motion of the bi-layered samples.

### 3.4. Physicochemical Investigation of Hydrogel Nanocomposites

**Figure 5:**
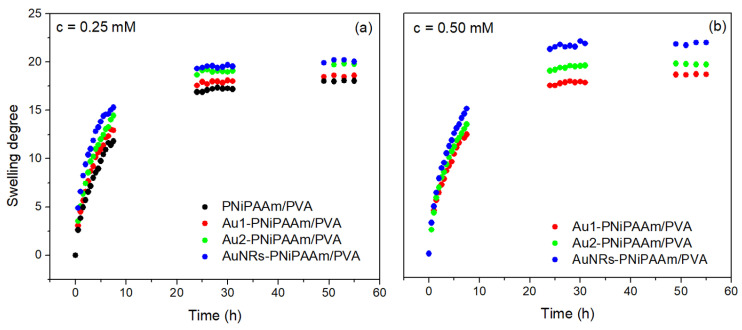
Swelling curves of bi-layered nano Au-PNiPAAm/PVA hydrogel nanocomposites with different concentrations of AuNPs: 0.25 mM (**a**) and 0.50 mM (**b**).

The physicochemical characterization of bi-layered nano Au-PNiPAAm/PVA hydrogel nanocomposites was conducted to assess their swelling/deswelling behaviors and the volume phase transition temperature (VPTT). The swelling kinetics and diffusion characteristics of the hydrogel nanocomposites were examined in distilled water under ambient conditions. Key parameters such as the swelling capacity and equilibrium swelling degree (*SD_eq_*) (Equation (2)) are crucial in determining the hydrogel’s potential applications, as they influence a variety of other properties. The swelling process is driven by the diffusion of water molecules inside the polymer matrix and their interaction with the functional groups on the polymer chains, causing it to expand. Swelling capacity is influenced by numerous factors, such as polymer composition, crosslinking density, pH and ionic strength, temperature, etc. The ability to manipulate hydrogels’ physicochemical features makes them extremely flexible materials for a variety of applications [8,29,30,31,32]. As shown in Figure 5 and Table 1, the incorporation of both spherical- and rod-shaped AuNPs into the active PNiPAAm layer causes an increase in the swelling capacity of bi-layered nano Au-PNiPAAm/PVA hydrogel nanocomposites compared to the pure bi-layered PNiPAAm/PVA hydrogel. The presence of nanoparticles generally increases the free space between polymer chains, causing the expansion of the polymer network and allowing the penetration of more water molecules, thus improving the network’s fluid absorption capacity.

Considering the obtained results, it can be summarized that the swelling capacity of bi-layered nano Au-PNiPAAm/PVA hydrogel nanocomposites is more strongly influenced by the shape than by the size or concentration of AuNPs incorporated into the PNiPAAm layer. In addition, the *SD_eq_* increases slightly with increasing concentration, and this effect is more pronounced for the smaller nanospheres (Au1, *d_av_* = 17 nm). Variations in the diameter of AuNSs only influenced the swelling capacity at lower concentrations (c = 0.25 mM). It is important to note that the increase in the *SD_eq_* is most pronounced for the bi-layered hydrogel nanocomposites with incorporated AuNRs, which show an increase of about 20.5% compared to the pure bi-layered PNiPAAm/PVA hydrogel.

The analysis of the fluid transport mechanism and the determination of the diffusion coefficient for every phase of the swelling process are essential for understanding the dynamic swelling mechanism. Due to the varying diffusion coefficient throughout the different fluid absorption stages, modeling the swelling process can be challenging. Considering that swelling is a dynamic process accompanied by changes in the fluid’s free volume and the fluid-polymer matrix ratio, a universal model of the fluid-polymer diffusion mechanism has yet to be developed. In this investigation, three various mathematical models were used to describe the swelling process and establish diffusion parameters. Kinetic parameters of diffusion process were determined by using empirical equation based on Fick’s law:

(4) $$\frac{SD}{{SD}_{eq}}=kt^{n}$$

where *k* is the kinetic rate constant related to the structure of network and penetrant, *n* is a characteristic exponent describing transport mode of the penetrant trough the polymer network and *t* is time [16,33,34]. In order to determine the diffusion coefficients, three approximations based on Fick’s law were used: the early-time model (Equation (5)), which describes the first 60% of the swelling process (*SD/SD_eq_* < 0.6), the late-time model (Equation (6)), which is valid for the last 40% of the swelling process (0.6 < *SD/SD_eq_ <* 1.0) and the Etters model (Equation (7)), which approximates the diffusion process for the entire swelling process (0 < *SD/SD_eq_ <* 1). The following equations describe the approximation models:

(5) $$\frac{SD}{{SD}_{eq}}=4{\left(\frac{D_{early}t}{\pi \delta^{2}}\right)}^{1/2}$$

(6) $$\frac{SD}{{SD}_{eq}}\;=1-\frac{8}{\pi^{2}}exp\left(-\frac{D_{late}\pi^{2}t}{\delta^{2}}\right)$$

(7) $$\;\frac{SD}{{SD}_{eq}}=1-exp{\left[-K{\left(\frac{D_{Etters}t}{\delta^{2}}\right)}^{a}\right]}^{1/b}$$

where *D_early_*, *D_late_* and *D_Etters_* are the diffusion coefficients of the medium for the early, late and entire range of swelling, respectively, *t* is the time, *δ* is xerogel thickness, and the constants for the Etters model are a = 1.3390, b = 2.6001 and K = 10.5449 [16,33,35]. The values obtained for characteristic swelling and diffusion parameters are presented in Table 1 and Table 2.

**Figure 6:**
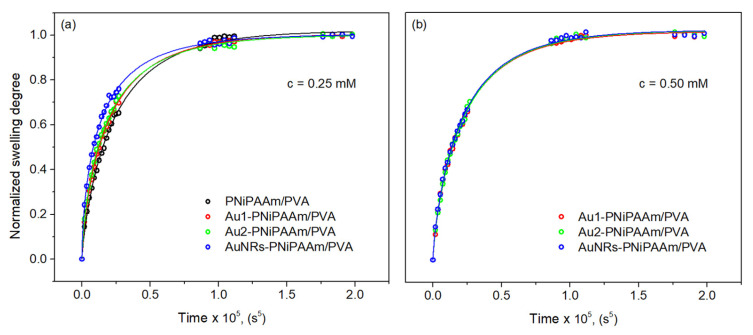
Etters approximation model for bi-layered nano Au-PNiPAAm/PVA hydrogel nanocomposites with different concentration of AuNPs: 0.25 mM (**a**) and 0.50 mM (**b**).

The logarithmic form of Equation (4) for the linear stage of the swelling process (approximately for the first 60%) allows an estimation of the diffusion exponent *n* and the dominant fluid diffusion model. Depending on the value of *n*, different models can be used to describe the diffusion: (i) *n* ≤ 0.5—Fick diffusion (dominant influence of fluid diffusion process), (ii) 0.5 < *n* < 1—non-Fick diffusion (comparable influence of polymer chain relaxation and fluid diffusion) and (iii) *n =* 1—case II diffusion (dominant influence of the polymer chains relaxation) [8,16,36]. For all investigated samples, the values of the diffusion exponent are in the range from 0.55 to 0.62 (Table 1), indicating that the swelling mechanism was influenced by both fluid diffusion and relaxation of polymer chains. It is evident that the different shapes, sizes and concentrations of nanoparticles have no influence on the swelling mechanism. On the other hand, the change in the applicability range of water sorption at various stages of swelling, as well as diffusion coefficients, were determined by fitting experimentally obtained data by using mathematical models (Equations (5)–(7)). The obtained diffusion coefficients, along with the corresponding correlation coefficients (R^2^), are presented in Table 2, while an example of the approximation model fits is shown in Figure 6.

The values of the diffusion coefficients for the applied mathematical models show that the diffusion coefficients are larger for samples with higher swelling capacity, which is expected and consistent with previous studies [33,35,37]. This tendency was observed for all three models, and it was most pronounced in the late phase of swelling. The obtained results indicate that the water diffusion rate is higher when the hydrogels are more hydrated, implying that the highest values were achieved for AuNRs-PNiPAAm/PVA hydrogel nanocomposites at both concentrations and during the final swelling phase. The diffusion coefficient values for all investigated samples were classified as follows: late-time model > Etters model > early-time model, indicating that Etters model “smoothed” the transition in the profile between the early and late diffusion times [33]. The water uptake into the hydrogel was slower in the beginning than it was in the middle and later stages of swelling [16,37]. Among the three applied approximations, the Etter model showed the best fit with the experimental results during the entire swelling process (has the highest R^2^ values). In addition, it is important to emphasize that the incorporation of both AuNSs and AuNRs leads to a slight expansion of the polymer network, resulting in a higher swelling capacity (larger *SD_eq_*) and a higher diffusion rate (higher diffusion coefficient), which is consistent with Reinhart and Peppas theory [38,39]. Furthermore, the obtained results revealed that all investigated bi-layered nano Au-PNiPAAm/PVA hydrogel nanocomposites show typical diffusion coefficient values for water diffusion in polymers (10^−9^–10^−8^ cm^2^/s), indicating generally rapid diffusion [35,40].

**Figure 7:**
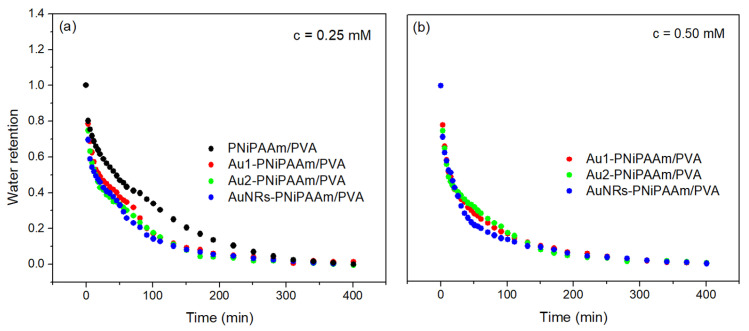
Deswelling curves for bi-layered nano Au-PNiPAAm/PVA hydrogel nanocomposites with different concentrations of AuNPs: 0.25 mM (**a**) and 0.50 mM (**b**).

To evaluate the thermal response of bi-layered nano Au-PNiPAAm/PVA hydrogel nanocomposites, due to the thermosensitivity of PNiPAAm as the active layer, the behavior of the investigated samples at temperatures above the VPTT was studied through the deswelling kinetics experiment. The water retention (Equation (3)) was determined gravimetrically, and the obtained curves are presented in Figure 7 [8].

The illustrated diagrams represent typical deswelling curves that describe the complete fluid release process until equilibrium is achieved. The deswelling rate (*r_d_*) can be described by the first-order kinetics equation:

(8) $$r_{d}=K_{d}\;(q_{t}-q_{md})$$

while the semi-logarithmic plot of Equation (8) was employed to fit the deswelling’s time dependence:

(9) $$ln\left[\frac{q_{t}-q_{md}}{q_{0}-q_{md}}\right]=-K_{d}\cdot t$$

where *K_d_* is the deswelling rate constant, *q_t_* is the mass of hydrogel at time *t*, *q_md_* is the mass of hydrogel at the end of the deswelling process, *q*_0_ is the weight of hydrogel before experiment (equilibrium swelling state), and *t* is time [16,41]. The obtained deswelling rate constants (Table 1) clearly show that the incorporation of nanoparticles into the PNiPAAm layer leads to an increase in the deswelling rate of bi-layered nano Au-PNiPAAm/PVA hydrogel nanocomposites. The *K_d_* values were significantly higher for hydrogel nanocomposites with incorporated AuNRs, indicating that this system should give the fastest and most pronounced response during nanoparticle actuation, i.e., the photo–thermal effect.

**Figure 8:**
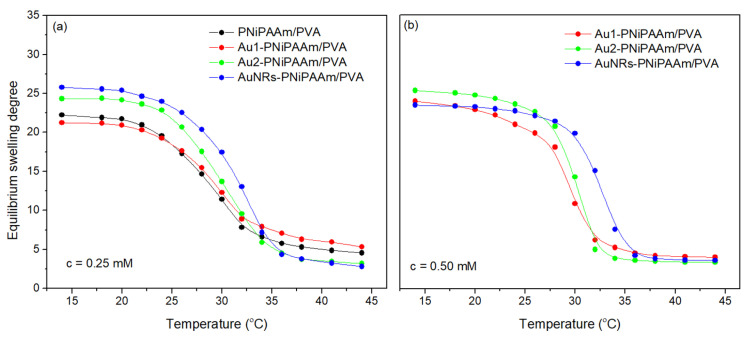
Temperature dependence of the *SD_eq_* for bi-layered nano Au-PNiPAAm/PVA hydrogel nanocomposites with different concentrations of AuNPs: 0.25 mM (**a**) and 0.50 mM (**b**).

Considering that the thermosensitivity of PNiPAAm, as an active layer, is a required property for achieving the intended mechanical response of bi-layered systems after excitation of nanoparticles and local temperature increase, the VPTT values of the tested systems must be precisely determined. Figure 8 illustrates the results of the gravimetric determination of VPTT values, which involved monitoring the equilibrium swelling degree in distilled water at temperatures ranging from 15 to 45 °C.

At temperatures below VPTT, PNiPAAm’s hydrophilic characteristic keeps the polymer chains hydrated, allowing the polymer network to expand and swell. On the other hand, at temperatures above VPTT, the hydrophobic interactions between the polymer chains become dominant, causing the shrinkage and collapse of the polymer network. Based on the results presented in Table 1, it can be concluded that the incorporation of AuNPs into the PNiPAAm layer leads to an increase in the VPTT of bi-layered nano Au-PNiPAAm/PVA hydrogel nanocomposites towards higher values compared to the pure PNiPAAm/PVA hydrogel. This effect is more pronounced in the case of nanorods, and it should be emphasized that the concentration of incorporated AuNPs does not have a significant influence. As already described in previous studies, this effect is expected, given the geometry of nanorods, which are more difficult to pack between polymer chains and cause a considerable expansion of the polymer matrix [6,7,8,42].

### 3.5. Structural Characterization of AuNPs Embedded into the Polymer Network

**Figure 9:**
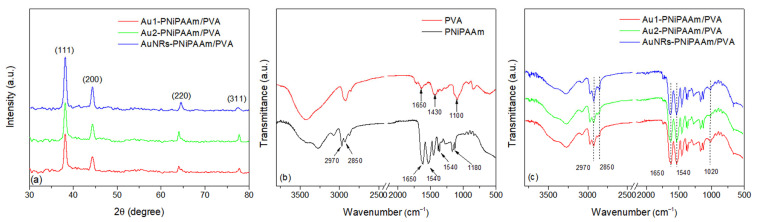
X-ray diffraction patterns (**a**) and FTIR spectra of PNiPAAm and PVA layer (**b**) and bi-layered nano Au-PNiPAAm hydrogel nanocomposites (**c**).

XRD analysis is a suitable method for structural investigation of nanoscale materials, and can give some insight into the crystalline structure, phase identification, sample purity, crystallite size and, in some cases, morphology [43]. Figure 9a shows a representative example of the XRD patterns for the tested bi-layered nano Au-PNiPAAm/PVA hydrogel nanocomposites, which contain a lower concentration of AuNPs in the active layer. Diffraction maxima at 2*θ* of approximately 38.3°, 44.6°, 64.6° and 77.9° correspond to the Bragg reflections from (111), (200), (220) and (311) lattice planes, respectively (JCPDS card no. 04-0784) [8,44]. These crystal planes represent the surface-centered cubic (*fcc*) structure of nanoparticles. Since the AuNPs were exposed to gamma irradiation during the formation of the PNiPAAm polymer network as an active layer, this method can provide information on how the gamma irradiation and the presence of the polymer network affect the crystallinity and stability of the nanoparticles. The intensity and sharpness of the observed peaks showed a high degree of crystallinity of the AuNPs, while no diffraction peaks of impurities were seen in the XRD patterns [45,46].

In order to determine the average crystalline domain size from diffraction peaks, Scherrer’s equation is used:

(10) $$D_{Sch}=\frac{k\;\cdot \;\lambda }{\beta \cos\theta }$$

where *k* is the Sheerer constant (typically 0.9), *λ* is a wavelength that depends on the type of X-rays used, *β* is the width (full-width at half-maximum) of the X-ray diffraction peak in radians, and *θ* is the diffraction angle. As long as the sample can be generally defined as a uniform, spherical particle, each peak may be examined individually and should provide a consistent crystalline domain size. In the presented calculation, the *D_Sch_* represents an average value obtained from all the presented diffraction peaks. The average crystalline domain sizes determined from these calculations were 18.1 nm and 31.3 nm for Au1-PNiPAAm/PVA and Au2-PNiPAAm/PVA hydrogel nanocomposites, respectively, with the initial sizes of AuNSs being 17 nm and 30 nm, respectively [43].

FTIR spectroscopy is a key technique for analyzing the chemical structure of hydrogels, allowing the identification of characteristic functional groups specific to the polymer. In the case of hydrogels, particularly those incorporating nanoparticles, FTIR is used to confirm crosslinking and detect functional groups such as amide, hydroxyl and carbonyl. It is important to note that AuNPs can influence the molecular structure of the hydrogel by changing hydrogen bonding, electrostatic interactions or coordination with functional groups. These changes are reflected as shifts in wave numbers and changes in the intensity of characteristic bands in the FTIR spectrum. For the examined bi-layered nano Au-PNiPAAm/PVA hydrogel nanocomposites, FTIR spectra were also recorded for PNiPAAm and PVA layers individually. The FTIR spectra of both PNiPAAm and PVA layers, as well as bi-layered nano Au-PNiPAAm/PVA hydrogel nanocomposites, are shown in Figure 9b,c, respectively.

The FTIR spectrum of the PVA layer reveals the following absorption features (Figure 9b). Notably, a broad band within the range of 3100–3650 cm^−1^ is attributed to the stretching vibrations of hydrogen-bonded –OH groups alongside the stretching of free hydroxyl groups. This broad absorption originates from the combined stretching of bonded (inter- and intramolecular hydrogen bonds) and non-bonded hydroxyl groups present in the PVA structure [47,48]. The absorption band near 2900 cm^−1^ corresponds to the overlapping asymmetric and symmetric stretching vibrations of the methylene groups. A weak band around 1650 cm^−1^ can be attributed to the stretching vibrations of residual carbonyl (C=O) groups from acetate units, arising from the specific degree of hydrolysis of the commercial PVA used in the synthesis [49]. However, its low intensity indicates that most monomers have been effectively crosslinked. The band observed near 1430 cm^−1^ arises from the bending vibrations of the CH_2_ groups, while the band at approximately 1100 cm^−1^ is associated with the stretching vibrations of the C–O bond. The observed bands confirm the successful synthesis and stable structure of the PVA layer [8]. The FTIR spectra of the PNiPAAm hydrogel and nano Au-PNiPAAm/PVA hydrogel nanocomposites exhibit distinct peaks corresponding to specific functional groups. The broad absorption band observed between 3700 and 3100 cm^−1^ corresponds to the N–H stretching vibration of the secondary amide group in PNiPAAm. The asymmetrical C–H stretching is observed at about 2970 cm^−1^, while the symmetric C–H stretching occurs at 2850 cm^−1^. The characteristic amide I band appears at 1650 cm^−1^, attributed to the C=O stretching of the amide group, while the amide II band is observed at 1540 cm^−1^, corresponding to N–H bending vibrations. Additionally, the spectra show –CH_2_ and –CH_3_ bending vibrations at 1450 cm^−1^ and 1380 cm^−1^, respectively, originating from the isopropyl group, along with C–N stretching around 1180 cm^−1^ (amide III band) [50,51].

The presence of AuNPs can be confirmed through changes in the intensity and position of polymer-specific bands in the FTIR spectrum, as they interact with functional groups within the polymer matrix. For instance, the decrease in the intensity of N–H stretching vibrations around 3300 cm^−1^ suggests that nanoparticles affect hydrogen bonding within the hydrogel. A decrease in the intensity of bands in the 2970–2850 cm^−1^ region, corresponding to asymmetric and symmetric C–H stretching vibrations of PNiPAAm, was observed in samples containing gold nanoparticles. This change can be attributed to donor–acceptor interactions between gold atoms and the amide groups of the polymer, which induce polarization of local bonds within the polymer chain and affect the vibrational behavior. The impact is most significant in samples with gold nanospheres, as their size and geometry enhance the interaction with the donor atoms in PNiPAAm. The most significant reduction is observed in samples containing AuNRs, indicating a stronger interaction between PNiPAAm and AuNRs compared to AuNSs. Additionally, a decrease in the intensity of the amide I band (~1650 cm^−1^) further indicates the interaction between nanoparticles and the electron-donating groups of the polymer (C=O). This effect is more pronounced with smaller AuNSs (17 nm) and AuNRs, probably due to their higher surface area and enhanced interfacial interactions, contributing to improved stabilization. Overall, the most significant spectral changes are observed for AuNR-PNiPAAm/PVA hydrogel nanocomposites, which show a decrease in the intensity of both the amide I band and N–H stretching vibrations. This suggests strong interactions between these nanoparticles and the polymer matrix. AuNSs of 17 nm in size exhibit a more significant effect compared to the 30 nm ones, which can be attributed to their smaller size, allowing for better stabilization and more effective surface interaction with polymer molecules [8,16]. It is also important to highlight the absorption bands observed in the 1000–1120 cm^−1^ region of the FTIR spectrum for Au-PNiPAAm/PVA nanocomposites, which are attributed to the C–N stretching vibrations of amide groups. In samples containing nanoparticles (Figure 9c), an increase in the intensity of these bands suggests stronger interactions between the AuNPs and the nitrogen atoms from the amide groups. This interaction is probably promoted by a donor–acceptor mechanism (N–Au), where the coordination between the nitrogen lone pair and the gold surface leads to bond polarization. This polarization enhances the dipole moment during the vibrational process, resulting in increased absorption intensity of the corresponding FTIR bands. The effect is more pronounced in the case of AuNRs, as their anisotropic shape allows for better integration into the polymer network. For AuNSs, the influence is more evident for 17 nm nanospheres, as their smaller size leads to higher surface energy, promoting stronger interactions with the polymer matrix. However, larger nanoparticles exhibit a more pronounced steric effect, decreasing stabilization and thus reducing their influence on the intensity of the FTIR bands [52,53,54].

### 3.6. Mechanical Properties of Hydrogels

**Figure 10:**
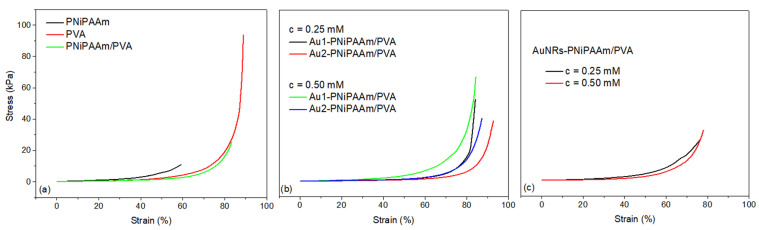
Stress–strain curves for investigated samples: PNiPAAm, PVA and bi-layered PNiPAAm/PVA hydrogels (**a**), bi-layered AuNS-PNiPAAm/PVA (**b**) and bi-layered AuNRs-PNiPAAm/PVA hydrogel nanocomposites (**c**).

A general characteristic of hydrogels, in their swollen form, is the possibility to instantaneously respond to low mechanical stress and then completely recover after the removal of external loads. In the case of bi-layered hydrogels, it is particularly important to emphasize the necessity of exhibiting adequate mechanical properties to prove the appropriate connection of the two layers and their ability to withstand the bending process without splitting and separation of the layers. A single static compression test was carried out at a strain rate of 2 mm/min, at room temperature, and characteristic stress–strain curves are presented on Figure 10. The compressive stress–strain curves for all the investigated samples show a typical exponential shape which is characteristic of an elastic polymer network in the hydrogel swollen state. When applying an external compressive force, the water is redistributed in the polymer matrix, and the material deforms and shows elastic behavior until the final rupture point is reached. The compressive modulus was calculated as the slope of the stress–strain curve from the linear region between 0% and 10% strain. At least three specimens were tested for each hydrogel, and the mean values and standard deviations were calculated [55,56]. The key parameters derived from mechanical testing (compressive strength (*σ_c_*), deformation capacity (*M_s_*) and the compressive elastic modulus (*E_c_*)) are summarized and presented in Table 3. Based on the results obtained for pure polymer matrices (Figure 10a), it can be concluded that the PNiPAAm hydrogel has the weakest mechanical properties, as it reaches the breaking point at a force of 10.7 kPa with a deformation of 59%. On the contrary, the PVA is a polymer known for its high elasticity and durability, possessing much better mechanical properties and, in the investigated case, withstanding a force of almost 100 kPa at a deformation of 90%. Upon examination of the bi-layered PNiPAAm/PVA hydrogel, it is evident that the addition of PVA as a passive layer enhanced the mechanical properties, which was the goal considering the potential applications of bi-layered systems.

Moreover, the incorporation of both AuNSs and AuNRs leads to an improvement in mechanical properties, given that nanoparticles within the polymer matrix act as additional junction points, thereby reinforcing the crosslinked network. The incorporation of nanoparticles results in a notable increase in the mechanical strength of bi-layer systems, which can endure higher stress (ranging from 39 to 67 kPa) while exhibiting nearly identical deformation (approximately 85%). It is essential to highlight that, in this case, the size of the AuNSs has a more significant influence on the material properties than their concentration. The best mechanical properties were observed for the bi-layered Au1-PNiPAAm/PVA hydrogel nanocomposites containing nanospheres with a diameter of 17 nm, and for both investigated concentrations. This finding was also confirmed by the highest values of *σ_c_* (52.7 and 67 kPa) determined for these samples, which are twice as high as the corresponding values for samples with incorporated AuNRs. This can be explained by the fact that smaller nanoparticles can pack more effectively between polymer chains, increasing the polymer matrix’s stability.

On the other hand, due to their geometry, the AuNRs are more challenging to pack inside the crosslinked polymer matrices, resulting in higher porosity and swelling capacity, as demonstrated in swelling studies. As a result, bi-layered AuNRs-PNiPAAm/PVA hydrogel nanocomposites exhibit poorer mechanical properties, indicated by only slightly higher compressive strength values compared to those of pure bi-layered PNiPAAm/PVA polymer matrix, especially at lower nanorod concentrations. Values of the modulus of elasticity under compression (*E_c_*) follow a similar trend, with the maximum values observed for the smaller Au1 nanospheres for both concentrations, while the samples with AuNRs showed much lower values.

### 3.7. Photo–Thermal Effect of Bi-Layered Nano Au-PNiPAAm/PVA Hydrogel Nanocomposites

**Figure 11:**
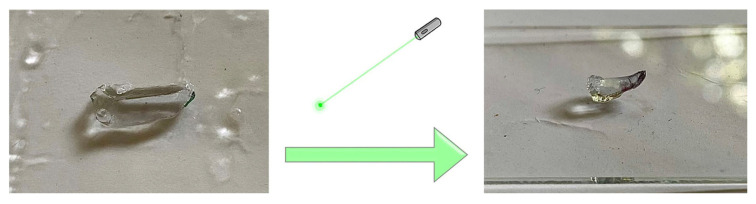
Photo–thermal effect induced by green laser irradiation on the AuNRs (0.25 mM)-PNiPAAm hydrogel nanocomposites as an active layer.

AuNRs-PNiPAAm hydrogel nanocomposite, as an active layer, was exposed to green laser irradiation in order to examine the photo–hermal effect. When a laser beam (wavelength: 520 nm, power: 200 mW) was focused on the middle of the square-shaped sample, the hydrogel bent due to volume shrinkage caused by the photo–thermal effect at the laser focus. The region irradiated by the green laser experienced an increase in local temperature due to the absorption of photonic energy by incorporated AuNR_S_. With an increase in temperature (above LCST), the hydrogen bonds between the hydrophilic amide groups and the water molecules are weakened, and the hydrophobic interactions among the isopropyl groups become strong. In this state, PNiPAAm polymer chains are dehydrated and aggregate into a tightly packed conformation, inducing the hydrogel to shrink and exclude water (Figure 11) [57,58].

After 60 s of the laser being turned on and focused on the hydrogel, the sample began bending in response to rising temperatures. By turning off the laser irradiation, the temperature of the hydrogel continuously decreased and the bending disappeared, thus allowing for reversible switchable movement. The whole bending process was visually observed and photographically recorded.

**Figure 12:**
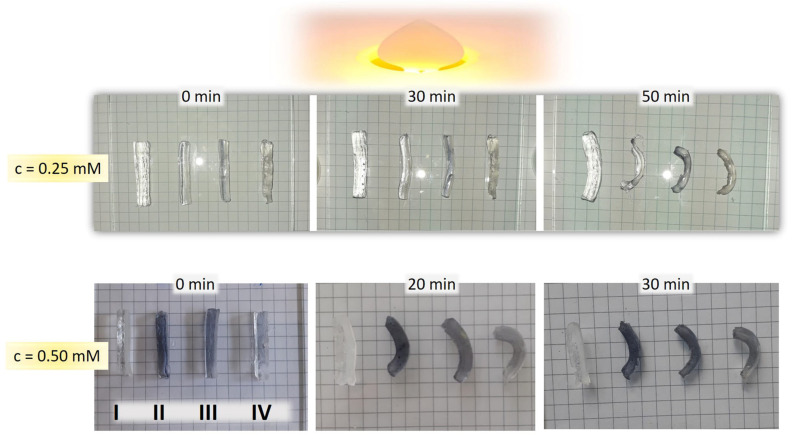
Photo–thermal effect induced by visible light irradiation on bi-layered nano Au-PNiPAAm/PVA hydrogel nanocomposites with different concentration of AuNPs. (Samples are labeled as I: PNiPAAm/PVA; II: Au1-PNiPAAm/PVA; III: Au2-PNiPAAm/PVA; and IV: AuNRs-PNiPAAm/PVA).

In order to achieve bending motion upon light exposure, the fully swollen bi-layered nano Au-PNiPAAm/PVA hydrogel nanocomposites were tested under visible light irradiation. When samples are irradiated by visible light, the photonic energy is absorbed by the AuNPs trapped in hydrogel network and rapidly converted into thermal energy. Therefore, the absorption of visible light leads to a remarkable deswelling of the PNiPAAm layer, as a thermo-responsive component, which is noticeable as mechanical deformation. To evaluate the effect of AuNPs, we conducted a control experiment on the photo-induced bending of a bi-layered PNiPAAm/PVA hydrogel without AuNPs. As can be seen in Figure 12, the bending as mechanical deformation is obvious for both AuNSs and AuNRs. Despite the higher value of VPTT for bi-layered AuNRs-PNiPAAm hydrogel nanocomposites, those systems show higher photo–thermal efficiency under visible light irradiation, inducing more pronounced bending deformation. Furthermore, the faster response was achieved for higher concentrations of AuNPs. These results indicate that it is possible to achieve applications of these types of nanocomposites in soft robotics and efficient energy conversion [12,59].

## 4. Conclusions

In summary, bi-layered nano Au-PNiPAAm/PVA hydrogel nanocomposites, with incorporated AuNSs and AuNRs, were successfully produced by a two-step synthesis process that combines freeze–thaw and radiolytic methods. The bi-layered hydrogel nanocomposites were obtained by gamma irradiation-induced crosslinking of PNiPAAm hydrogel in the presence of AuNPs (active layer), over the frozen PVA hydrogel obtained by the freeze–thawing method (passive layer). The influence of different shapes, sizes and concentrations of AuNPs on optical, physicochemical, structural and mechanical properties of bi-layered hydrogel nanocomposites was investigated, as well as their photo–thermal effect. The TEM and XRD analysis confirmed the formation of AuNSs with a mean diameter of around 17 nm and 30 nm, and AuNRs with an aspect ratio of around 4.5 (diameter ≈ 10 nm and length ≈ 45 nm), both with the face-centered cubic crystal structure. The physicochemical parameters of bi-layered nano Au-PNiPAAm/PVA hydrogel nanocomposites indicate that the incorporation of AuNRs had a greater influence in comparison to AuNSs, causing higher swelling capacity, diffusion coefficients and VPTT values. Furthermore, the mechanical properties of bi-layered hydrogel nanocomposites were improved by the incorporation of both AuNSs and AuNRs, which act as additional strengthening points of the polymer network. The best mechanical properties are possessed by the bi-layered hydrogel nanocomposites with the smaller AuNSs because smaller NPs can pack more effectively between polymer chains, thus increasing the polymer matrix’s stability. Finally, the photo–thermal effect of bi-layered nano Au-PNiPAAm/PVA hydrogel nanocomposites was observed. Namely, under visible light irradiation, the photonic energy absorbed by the incorporated AuNPs is rapidly converted into thermal energy, causing local heating and shrinkage of the active layer. Along with the contraction of the active layer, there is also a displacement of the passive layer, which together leads to mechanical deformation demonstrated as bending. The higher photo–thermal efficiency and more pronounced bending deformation were noticed for hydrogel nanocomposites containing AuNRs. This bending response reveals the huge application potential of the investigated materials as soft actuators.

## Figures and Tables

**Table 1:** Physicochemical parameters of bi-layered nano Au-PNiPAAm/PVA hydrogel nanocomposites.

Sample	c(Au) (mM)	*d_av_* (nm)	*SD_eq_*	*n*	*K_d_* × 10^2^ (1/min)	VPTT (°C)
PNiPAAm/PVA	0		18.1 ± 0.2	0.57 ± 0.01	1.96 ± 0.08	29.5 ± 0.5
AuNSs-PNiPAAm/PVA	0.25	17	18.5 ± 0.3	0.57 ± 0.02	2.18 ± 0.07	29.6 ± 0.6
		30	19.3 ± 0.4	0.55 ± 0.02	2.52 ± 0.08	30.1 ± 0.5
	0.50	17	19.5 ± 0.5	0.62 ± 0.03	2.33 ± 0.08	29.7 ± 0.4
		30	19.6 ± 0.3	0.61 ± 0.01	2.49 ± 0.09	30.0 ± 0.5
AuNRs-PNiPAAm/PVA	0.25		20.1 ± 0.5	0.56 ± 0.01	4.10 ± 0.11	32.0 ± 0.7
	0.50		21.8 ± 0.6	0.58 ± 0.02	4.30 ± 0.12	32.3 ± 0.6

**Table 2:** Values of the diffusion coefficient obtained by swelling approximation models.

Sample	c(Au) (mM)	*d_av_* (nm)	*D_early_* × 10^8^ (cm^2^/s)	R^2^	*D_late_* × 10^8^ (cm^2^/s)	R^2^	*D_Etters_* × 10^8^ (cm^2^/s)	R^2^
PNiPAAm/PVA	0		2.50 ± 0.11	0.99	3.40 ± 0.16	0.98	3.35 ± 0.09	0.98
AuNSs-PNiPAAm/PVA	0.25	17	3.12 ± 0.15	0.98	3.81 ± 0.19	0.97	3.63 ± 0.11	0.99
		30	3.37 ± 0.17	0.99	3.97 ± 0.20	0.98	3.85 ± 0.12	0.99
	0.50	17	3.25 ± 0.16	0.99	5.12 ± 0.23	0.97	3.55 ± 0.10	0.99
		30	3.89 ± 0.28	0.97	3.56 ± 0.18	0.99	3.61 ± 0.14	0.99
AuNRs- PNiPAAm/PVA	0.25		3.61 ± 0.17	0.96	4.88 ± 0.21	0.98	4.62 ± 0.13	0.98
	0.50		3.83 ± 0.20	0.98	4.91 ± 0.22	0.96	4.89 ± 0.16	0.99

**Table 3:** Mechanical parameters of bi-layered nano Au-PNiPAAm/PVA hydrogel nanocomposites.

Sample	c(Au) (mM)	*d_av_* (nm)	*σ_c_* (kPa)	*M_s_* (%)	*E_c_* (kPa)
PNiPAAm	0		10.7 ± 0.4	59.0 ± 2.6	1.31 ± 0.08
PVA			93.8 ± 3.9	88.9 ± 3.4	2.72 ± 0.15
PNiPAAm/PVA			25.3 ± 1.1	83.3 ± 2.9	1.90 ± 0.13
AuNSs-PNiPAAm/PVA	0.25	17	52.7 ± 2.8	84.3 ± 3.6	3.21 ± 0.19
		30	38.9 ± 1.6	92.9 ± 3.8	1.33 ± 0.11
	0.50	17	67.0 ± 3.2	84.4 ± 3.1	4.01 ± 0.23
		30	40.5 ± 2.6	87.4 ± 3.0	2.10 ± 0.14
AuNRs-PNiPAAm/PVA	0.25		26.3 ± 1.2	76.5 ± 2.5	2.14 ± 0.12
	0.50		32.7 ± 2.1	78.1 ± 2.8	1.81 ± 0.11

## Data Availability

The original contributions presented in the study are included in the article; further inquiries can be directed to the corresponding author.
